# The Connection between Absorptive Capacity and Green Innovation: The Function of Board Capital and Environmental Regulation

**DOI:** 10.3390/ijerph20043119

**Published:** 2023-02-10

**Authors:** Sohail Ahmad Javeed, Boon Heng Teh, Tze San Ong, Nguyen Thi Phuong Lan, Saravanan Muthaiyah, Rashid Latief

**Affiliations:** 1 Management School, Hunan City University, Yiyang 413000, China; 2 Faculty of Management, Multimedia University, Persiaran Multimedia, Cyberjaya 63100, Malaysia; 3 School of Business and Economics, Universiti Putra, Serdang 43400, Malaysia; 4 Department of Business Administration, Daffodil International University, Dhaka 1341, Bangladesh; 5 School of Finance, Xuzhou University of Technology, Xuzhou 221018, China

**Keywords:** green innovation, absorptive capacity, board capital, environmental regulations

## Abstract

The stress of environmental regulations, sustainable development objectives, and global warming is becoming more prominent now. Most studies conclude that the industrial sector is largely at fault and under tremendous pressure to address these climate change issues. This study highlights the significance of green innovation to Chinese firms in mitigating these conservational challenges, and the study probes the association between green innovation and absorptive capacity. Additionally, board capital (the social and human capital of directors) and environmental regulation—both drivers of green innovation—are explored as moderators between green innovation and absorptive capacity. With appropriate econometric methods and theoretical support from the natural resource-based review, the resource dependency theory, and the Porter hypothesis, the results indicate the positive relationship between green innovation and absorptive capacity. They also reveal board capital and environmental regulation as positive moderators, emphasizing their significance to green innovation. This study offers several suggestions and directives for stakeholders, such as businesses, policymakers, and governments, to foster green innovation for greater profitability, minimizing negative industrial consequences.

## 1. Introduction

Authorities are motivated by how firms conduct their environmental activities and their contribution to environmental cleanup [1]. Environmental protection is constantly at risk due to the massive rise in firms’ manufacturing processes, greenhouse gas emissions, and resource exploitation. The United Nations Sustainable Development Goals (SDGs) [2], a shared blueprint that strives for peace and prosperity while protecting the environment, is primarily directed at the industrial sector [3]. The context of emerging economies is essential to this study because businesses in developing nations place a premium on environmental concerns.

Rafique et al. [4] claim that although the industrial sector boosts a country’s economic growth, it has left a negative impact on the environment. Environmental degradation has severely impeded economic progress, particularly in China. China’s economy is growing exponentially; however, it has not been without cost to the environment [5]. Faced with global criticism and scrutiny, Chinese firms want to improve environmental performance. Green innovation, along with other social practices, has gained emphasis [6]. The Chinese government has initiated steps to address the issue, which has been brought about mainly by the industrial sector [7]. They have adopted several measures to inspire a firm’s social practices, green strategies, and other conservational activities [8].

Green innovations are new manufacturing, management, or service models that mitigate environmental issues and have gained significant relevance [9]. It is an essential tool to accomplish environmental goals tactically. The advantages of green innovation have been thoroughly studied, but there needs to be more research to fully understand the variables that affect the firms that have invested in it [7]. Albort-Morant et al. [10] highlight the value of absorptive capacity in driving green innovation. They demonstrate that through external knowledge, firms become aware of their activities’ environmental detrimental impact, which convinces them to integrate green innovation strategies into their operations. Further, Chen et al. [11] argue that absorptive capacity positively impacts green radical, incremental, and service-based innovation.

The above prompts the following question: If a firm’s green innovation practices can be strengthened by absorptive capacity, what are the main factors that support this positive association? The study suggests using two key elements—board capital and environmental regulation—to boost absorptive capacity and green innovation.

First, enhanced board monitoring and guidance can be crucial for green innovation [12]. A board’s role will likely be amplified for environmental performance because green innovation is more challenging than conventional innovation. Allemand et al. [13] disclose that firms wanting to promote innovation are likelier to choose board members with the appropriate training and educational background. Additionally, Hughes et al. [14] mention that a firm can leverage absorptive capacity to transform the external knowledge it obtains from board capital into a valuable internal resource for performance enhancement. Board capital is the human and social capital of a firm’s directors [15].

Second, environmental regulations can promote green innovation, associating it with absorptive capacity. Such regulations can stimulate firms to develop unique products for the market, fostering innovation [5]. Jaffe et al. [16] note that rules encouraging green innovation push firms to be distinctive in the market. Because they are more prone to produce pollution, manufacturing firms adhere to environmental regulations more strictly and prioritize green innovation [17]. Moreover, regulation is essential for boosting a firm’s absorptive capacity [18]. They can pressure firms into doing research and development (R&D) on green innovation [19].

Third, this study is likely the first to explore board capital and environmental regulation as moderators of the association between absorptive capacity and green innovation. The natural resource-based review, the resource dependency theory, and the Porter hypothesis provide the theoretical support for this investigation.

A well-known objective of R&D is the creation of new products, services, and technology. The natural resource-based view incorporates the idea that a firm’s environmental skills and resources may offer proactive rather than reactive solutions to sustainability-related issues, reflecting the firm’s sustainability competence [20]. The resource dependency theory explains the importance of absorptive capacity for green innovation and other R&D actions [21]. The Porter hypothesis supports the role of internal capabilities, environmental responsibilities, and the senior executive in promoting corporate social and environmental actions [22].

This study sought data for 2010–2020 from all non-financial A-listed Chinese firms trading on the Shanghai and Shenzhen Stock Exchanges. Analysis was achieved using the fixed effect, GMM, and FGLS models. This study demonstrates how absorptive capacity is essential for green innovation. It also relates how board capital, namely human and social capital, and environmental regulation enhance green innovation. More importantly, this study argues that board capital favorably modifies the association between absorptive capacity and green innovation, suggesting that board capital can alleviate environmental challenges at the organizational level. It also affirms that environmental regulation positively moderates the association between absorptive capacity and green innovation. Governments and regulatory agencies can use the results to mitigate unfavorable industrial effects on the environment.

This study makes a theoretical contribution by adhering to the natural resource-based view, resource dependency theory, and the Porter hypothesis. This study offers potential means to achieve the UN’s sustainable development goals.

### Environmental Policy in China

The greatest threat to humanity is climate change brought on by greenhouse gas emissions, and CO_2_ emissions are the primary driver [23]. China introduced regulations to mitigate this threat in line with 1997’s Kyoto Protocol [24]. The country’s Five-Year Plans (FYP), which is its most significant mandated goal system, include top-down target-based environmental management such that local governments are tasked with meeting environmental criteria for unit pollution emissions and energy consumption. It provides the fastest and most effective means to achieve results under China’s vertical performance appraisal system.

The Two Control Zones (TCZ) policy, the first acknowledged target-based air pollution control strategy in history, has been extensively studied as a quasi-natural experiment to investigate the effects of China’s environmental regulations on pollution reduction, economic growth [25], infant mortality [26], and foreign direct investments [27]. The Key Cities for Air Pollution Control (KCAPC) concept was first put forth in an official document (known as the “two compliance policy”) by the Ministry of Environmental Protection (MEP) in 1998 to enhance the air quality in several significant cities. As the first batch of cities in the KCAPC, the central government chose 47 prefecture-level cities. The majority were municipalities (such as Beijing, Shanghai, Chongqing, and Tianjin), provincial capital cities, cities in special economic zones, popular tourist destinations, and coastal open cities.

The Law for the Prevention and Treatment of Air Pollution significantly selected these 66 cities. The process took place in 2000, involving a thorough economic analysis and assessment of the air pollution levels. In December 2016, the National People’s Congress passed China’s first Environmental Protection Tax Law. The Law, which took effect on 1 January 2018, establishes the rules for taxing entities that create noise pollution, solid waste, or air or water pollutants.

Even with regulation, implementing and adhering to China’s environmental standards have frequently impeded advancement. Although ambitious goals necessitate enhanced regulatory standards, monitoring, and policy implementation, the process is highly organic. Depending on their industry and scope, firms must adopt a manner of agility to minimize the risks associated with sudden changes. For firms to lawfully function in a particular jurisdiction, they must comply with environmental regulations, standards, and other requirements, an act known as green or environmental compliance. Given the government’s commitment to making China a green, low-carbon, and circular economy, green compliance will become more crucial for firms operating there.

After repeated delays, China finally opened the biggest carbon trading market on 16 July 2021. At its inception, the carbon market included more than 2225 power-producing companies, mostly state-owned enterprises (SOEs). These firms account for ten (10) to fourteen (14) percent of global emissions and over half of China’s emissions. The Ministry of Ecology and Environment (MEE) and its local counterparts established the “Enterprise Environmental Information Legal Disclosure System” on their official government websites to require firms to disclose and upload environmental information annually in a “Legal Disclosure Report of Environmental Information”.

The “pollutant discharge permit” also enables firms to release pollutants into the environment. Since mid-2010, China has gradually encouraged firms with the pollutant discharge permit system. Fourteen (14) authorities, including the MEE and the Supreme People’s Court, established the Rules on Compensation for Ecological and Environmental Damage on 26 April 2022. The rules specify the polluter’s obligations for reparable and irreparable harm.

**Figure 1:**
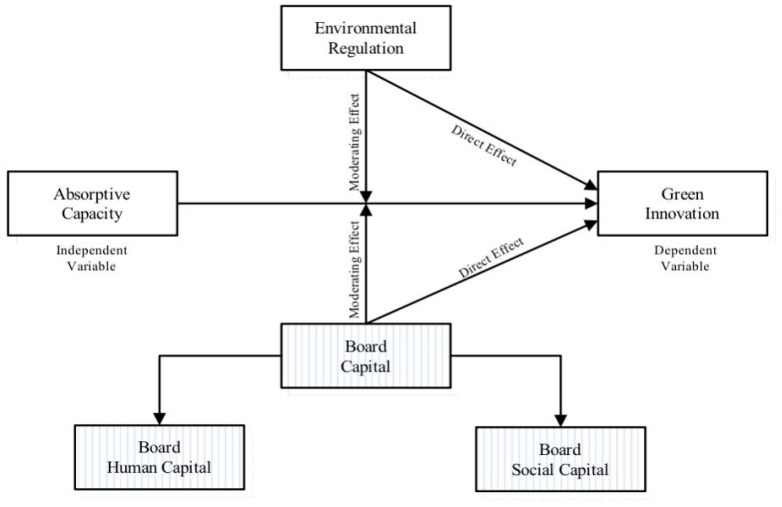
Theoretical Model.

The following section presents the empirical investigation and theoretical evaluation of the emerging hypotheses. The procedures for acquiring information, measuring variables, and conducting research are covered in Section 3. In this study’s Section 4, the results are reported. The conclusions, consequences, limits, and suggested next steps are accessible in Section 5. Figure 1 outlines the study’s setting.

## 2. Theoretical Base

### 2.1. Natural Resource-Based View (NRBV)

Business environmental initiatives are essential because they can improve operations while giving firms a competitive edge. The natural resource-based view gives insight into firms’ behavior when motivated to preserve the environment [20]. The theory’s three key objectives are long-term growth, responsible product use, and pollution prevention techniques. Different organizational skills resulting from a firm’s focus on natural resources give rise to different forms of efficient adoption of green innovation [28].

The resource-based perspective proposes that an organization can achieve competitiveness by using strategies that are elementally valuable, rare, inimitable, and organized (VIRO) [23]. Hart [20] expanded the term “resources” in NRBV. An example of a VIRO resource is the use of organizational knowledge to improve waste management procedures [24]. NRBV is a tool that supports a firm’s capacity to develop new VIRO resources and respond to complex demands and environmental issues. Considering how it reacts to global environmental changes, a firm may thus establish a sustainable competence.

Absorptive capacity is described explicitly by Cohen and Levinthal [25] as “the ability of the company to locate, incorporate, and utilize knowledge”. The learning happens because of a firm’s R&D investments in developing new technologies, goods, and procedures. A firm’s sustainability competence reflects a range of in-house environmental expertise and resources that may provide proactive rather than reactive responses to sustainability-related problems [24]. Researchers emphasize the significance of a firm’s capabilities, as they may affect the adoption of green innovations [28]. A firm’s absorptive capacity displays its potential to take in, recognize, and apply outside knowledge. Absorptive capacity has become increasingly important in fostering innovative performance and competitive capabilities [26].

### 2.2. Resource Dependency Theory

When firms are unsure about managing stakeholders’ new demands, there lies a powerful incentive to go outside the organization for resources. Pfeffer and Gerald [27] developed the resource dependence theory, explaining that firms can design structural configurations to acquire resources for internal processes. These structural measures are frequently relational and enable the transfer of knowledge, skills, and competencies from one organization to another. The capacity of a firm to absorb knowledge is absorptive capacity.

However, as Cohen and Levinthal [25] pointed out, developing the capacity to transform external knowledge for internal use was similar to the goal of R&D. Watson et al. [21] explained the importance of dynamic and operational capabilities to accomplish environmental results in this context. They claimed that engaging stakeholders would be a dynamic competence because the interaction could stimulate green innovation discussions and more. Operational capabilities are a product of learning from interactions with people outside the firm and are later disseminated across its various functional areas [29]. R&D investments may result in the creation of operational capabilities and, together with dynamic capabilities, help a firm achieve its environmental objectives.

Many firms remain uncertain of responding to stakeholder expectations for environmental sustainability, regardless of the intensified pressure to improve the environment since the late 1980s. According to Kiron et al. [30], a firm’s sustainable practices are the imperative of the chief executive officer (CEO). This conclusion implies that there needs to be more clarity in firms when it comes to environmental sustainability. Hence, the option lies in relying on sources outside the organization to decide how to proceed.

This study provides a case for both the board’s role and investing in green innovation by merging the ideas of resource dependency and absorptive capacity. Absorptive capability describes converting external information acquired through board capital into a functional internal reserve for performance enhancement [31]. Although board capital is the social capital in transferring complex knowledge among firms, Hughes et al. [14] suggest that individual firms may need more internal ability to learn from potential collaborating firms. External knowledge must be absorbed to be useful in enhancing performance.

In addressing the above, it is imperative to identify the appropriate board composition that makes for board capital. A firm is likely to create opportunities for knowledge acquisition from other firms. The absorptive capacity develops once this knowledge is implemented internally through R&D allocation. The implication is that internal routines and procedures collaborate with external structural arrangements to generate capacities to respond to stakeholder expectations for environmental performance.

### 2.3. Porter Hypothesis

Porter [22] made a case for how environmental regulations and modern company strategies to gain competitive advantage go hand in hand. He suggests that a firm can gain a first-mover advantage through environmental regulations and other social practices [22,32]. He emphasizes the significance of innovative and environmentally friendly corporate practices. Ambec et al. [33] reveal how environmental regulations can boost shareholder value, serving as an essential tool for innovation and competitive advantage. Porter stresses the significance of environmental regulations for firms seeking to gain a competitive edge through R&D. This study proposes environmental regulations as the external component to enhance R&D and green innovation. It can lead to a distinction between firms and contribute to a firm’s sustainability. It can encourage firms to participate in environmental cleanup efforts and adopt innovative sustainable practices.

### 2.4. Literature and Hypothesis Construction

#### 2.4.1. The Role of Absorptive Capacity

Cohen and Levinthal [31] pioneered absorptive capacity; it has undergone substantial conceptual and methodological development since then [34]. Absorptive capacity is the capability of an organization to understand the importance of obtaining, integrating, and using new external information [31]. It includes a firm’s potential to connect and incorporate this new external information with its prior knowledge. A firm’s ability to absorb new information is crucial in its capacity to create new ideas, products, and services with external knowledge [35], setting it apart from rivals [36] and leveraging it in terms of knowledge attainment [37].

Gluch et al. [38] define absorptive capacity as an organizational competence that improves green innovation consequences. Earlier studies suggest that adopting innovative techniques in the manufacturing or service context requires a firm to be able to gather, share, and utilize internal and external information [39]. They also highlight that firm innovation is achieved through absorptive capacity or knowledge-sharing. Albort-Morant et al. [10] argue that a firm’s ability to absorb new ideas improves its potential to develop green innovations, such as eco-friendly goods, facilities, and working practices. Firms will implement green innovation methods into their operations when they have regular access to external information and become aware of their operations’ damaging environmental effects.

Aboelmaged and Hashem [28] opine that knowledge sharing, as an absorptive capacity and key to the success of green innovation, is significantly impacted by leaders’ capacity to acquire internal and external information. Such a measure entails creating new ecologically friendly goods and methods for recycling, decreasing waste, and preventing pollution. Firms must embrace internal and external expertise to seek new ecologically friendly processes and address environmental issues. Chen et al. [11] used structural equation modeling to examine how green services provided by the Taiwanese electronics sector relate to absorptive capacity. Their results indicate that absorptive capacity positively affects green radical, incremental, and other innovations.

More external information and expertise are encouraged for green innovation [40]. Effectively utilizing external knowledge is crucial for boosting a firm’s situation into viability [41]. Firms must combine their internal expertise with external knowledge as a green innovation strategy. Absorptive capacity facilitates a firm’s capacity to absorb and integrate external knowledge at the organizational level [25,42]. It will likely accelerate the frequency, speed, and scale of a firm’s green innovation practices [43]. A firm’s ability to absorb external forces allows it the freedom to select the best course of action [34]. Implementing green innovation practices requires the management of substantial amounts of internal and external knowledge, frequently originating from many areas. It is necessary to internalize external knowledge, combine it with past relevant internal knowledge, and alter it. Therefore, firms must build their capacity to learn new information [44].

Hence, this study posits the following hypothesis:

**H1.**  *Absorptive capacity is advantageous for enhancing green innovation*.

#### 2.4.2. The Role of Board Capital

Green innovation requires high-level support because of its complex and protracted process [35]. Boards can provide effective oversight and direction in this matter [12]. The board’s knowledge of green innovation is crucial, given that green innovation is more complex than conventional innovation. This study contends that board capital encourages green innovation through training, experience, and board financing. Board capital is the human and social capital of board directors.

The human capital of a board enables better recommendations because the board members have access to knowledge configurations that make them understand challenging content [36]. Directors with advanced degrees significantly boost an organization’s effectiveness. Directors with extraordinary expertise are aware of strategic opportunities and are likelier to pay special attention to them [37]. Allemand et al. [13] assert that firms wishing to foster innovation are likelier to recruit board members with specific education and experience criteria. Education, experience, and talent are traits that can boost business innovation.

Board interlocks are one feature of the board structure that provides room for novel concepts. A board interlock happens when a director of one firm sits on the board of directors of another. Board members affiliated with other businesses may offer possibilities for knowledge acquisition by participating in other boards. The social capital of a board provides fresh perspectives and prospects for environmental development [45]. Jing Lu et al. [39] provide evidence that supports the role of social capital in enhancing green innovation. Social capital improves environmental outcomes [38].

Board capital enables enhanced knowledge, better strategic direction, more robust financial, social, and environmental performance, increased innovation, and efficient monitoring [46]. Board capital, similar to interlocking directorates, encourages green innovation [29].

De Villiers et al. [47] conducted an experimental study with data from U.S. companies to exemplify how additional directorships enhance the board’s ability to give guidance when dealing with more challenging strategic issues, such as environmental disasters. They also assert that social interaction can encourage sharing of beneficial resources and foster creativity. Building social and human capital, particularly in environmental enactment, is crucial for boosting corporate social responsibility (CSR) [48]. Board capital improves board monitoring, lessening management entrenchment and improving the success of green innovation. Board members with knowledge of conservational practices can lead green innovation at the organizational level [15].

Hence, this study posits the following hypothesis:

**H2.**  *Board capital enhances the green innovation of the firms*.

Environmental sustainability has adopted the notion of absorptive capacity. Zahra and George [44] reveal that absorptive capacity is essential for green innovation [49]. R&D investments may result in the creation of operational capabilities; along with dynamic capabilities, they can support a firm’s environmental goals. Although board capital can provide access to resources, whether a firm can use the information to change internally remains to be seen. Studies with survey methodologies have linked absorptive capacity to green innovation at the organizational level. According to Xie et al. [50], Chinese manufacturing-listed companies with robust absorptive capacity, as evaluated by R&D expenditure, are more aggressive in implementing process improvements relevant to the environment, for example, cleaner production.

Ingenbleek and Dentoni [51] came to a similar conclusion in their study of agribusiness in the Netherlands. They identify that stakeholder embeddedness is more successful in influencing the firm toward innovating corporate social responsibility than stakeholder pressure. This conclusion can be applied to board capital, implying that it is likelier to bring about change than institutional pressure.

Research indicates that internal management mechanisms are essential to realizing absorptive capacity; this is consistent with the findings of Zahra and George [52]. According to Watson et al. [21], increasing creative capacities through moderation with external stakeholders and board resources can improve environmental performance. Additionally, absorptive capacity helps a firm turn external knowledge into a valuable internal resource for performance improvement through board interlocks [14]. According to Chen [53], board capital advances the assessment of competing R&D projects. Higher absorption capacity facilitates internal transformation and exploitation of knowledge [54]. Top management support and R&D are essential in realizing absorptive capacity [55]. Absorptive capacity enables opportunities for knowledge acquisition and assimilation to address conservational concerns.

Hughes et al. [14] report even if board capital serves as social and human capital to transfer complex knowledge among businesses, some organizations may not have the internal resources to benefit from the experiences of other businesses with which they have connections. To be useful in enhancing performance, external knowledge must be absorbed. In order to address the dependence of board capital on others for improving environmental performance, board capital is strategically intended to provide opportunities for knowledge acquisition from other businesses, which operationalizes the board role [29]. However, unless internal R&D spending is used to put this information to use, the absorptive capacity does not develop. As a result, internal routines and procedures collaborate with structural arrangements that reach outward to generate energetic and operational capacities in order to respond to shareholder expectations for environmental performance [31].

According to Zou et al. [55], environmental process improvements, such as cleaner production, are aggressively implemented by Chinese manufacturing listed companies with stronger absorptive capacity, as indicated by R&D expenditure. According to an application of this finding to board capital, institutional pressures may not be as successful at bringing about change as is social and human capital built for acquiring external knowledge.

In order to solve environmental issues, board capital creates chances for knowledge acquisition and absorption from other businesses. Zona et al. [56] find that when a resource-constrained firm engages with a resource-rich firm, the engagement enhances the board capital of the former. As a result, performing firms in dependent industries are likelier to produce innovative practices, strategies, and expertise than those with average or subpar performance. Furthermore, firms become more confident in their ideas, technologies, and procedures regardless of the industry because of their enriched board capital. As a result, board capital with leaders may be more effective than that with those who perform less admirably. Board capital improves a firm’s absorptive capacity professionally and intellectually [39].

This study offers the following hypothesis:

**H3.**  *Board capital positively moderates the association amid Absorptive Capacity and Green Innovation*.

#### 2.4.3. The Role of Environmental Regulations

In addition to significant losses in environmental health, rapid economic expansion has been accompanied by declining environmental quality and resource degradation. Some firms have started incorporating environmental issues into their business plans in response to intense environmental pressure [57]. Environmental regulations significantly influence a firm’s policy actions because of the social game [45]. They motivate firms to engage in social practices that foster green innovation. Environmental regulations influence firms’ green innovation favorably [58], whereas absorptive capacity increases the institutional impact on green innovation. Institutional isomorphism is the process of coordinating a firm’s strategy and actions with the expectations of institutions [59]. They help firms reduce their adverse industrial effects, ultimately enhancing their reputations [57]. In addition, influential stakeholders can compel firms to adhere to multiple regulations given that the government and industry associations are the source of these regulatory restrictions [58].

Researchers have explained the connection amid environmental regulations and green innovation [45,57,59]. The concept of green innovation has also been generated through environmental practices. These regulations encourage companies to move toward green innovation [58]. Numerous studies demonstrate that firms’ efforts to establish legitimacy become increasingly comparable as institutional pressure grows. Environmental regulations force firms to improve or safeguard their legitimacy [34]. Due to environmental pollution issues, businesses have recently been in the news. Therefore, the importance of environmental regulations has also been enhanced [5]. Regulators implement measures to make firms pay the price for environmental degradation such as pollution [33], but these practices ultimately improve green innovation [60]. Firms implement proactive environmental strategies to reduce their environmental effect, obtain governmental backing, and establish legitimacy in the face of mounting pressure from increasingly stringent environmental legislation [61].

The broad notion that a firm’s environmental performance is desirable of prevalent expectations is environmental legitimacy, a subset of organizational legitimacy [62]. Porter et al. theorize that firms are pushed toward innovation by orchestrated environmental constraints through first-mover advantage and innovation compensation [63]. Environmental regulations force a firm to develop distinctive products for the market and a reputation for itself [33]. These views emphasize how environmental regulations assist firms in growing creatively and profitably.

Environmental regulations motivate firms to look for innovative ways to reduce the cost of compliance [64]. Barbera et al. [50] assert that environmental constraints increase firm productivity and market standing. Jaffe et al. [16] and Porter et al. [63] observe that environmental restrictions encourage green innovation for firms to thrive in a cutthroat market. More prone to generating pollution, manufacturing firms rigorously adhere to environmental regulations and prioritize green innovation [17].

Porter et al. [63] argue that environmental limitations provide businesses in a cutthroat market with a win–win situation by encouraging them to develop distinctive and innovative products for green invention. Previous studies disclose that environmental regulations are essential for promoting environmentally responsible conduct and reducing the negative consequences of business [51]. They can support business green innovation, according to this study. Unfavorable environmental issues have forced the focus on green and sustainable value creation, but doubts persist over whether sustainable development techniques can allay these concerns while enhancing sustainability and competitiveness [15]. According to Ma et al. [65], environmental legitimacy is crucial in associating institutional settings with green innovation.

This study provides the following hypothesis:

**H4.**  *Green innovation is influenced by environmental regulations*.

Absorptive capacity is the ability to absorb, understand, integrate, and modify external knowledge [31]. Previous research dictates that having absorptive capacity enables one to perceive environmental constraints and comprehend the mechanisms involved in overcoming green inertia [66]. Absorptive capacity is highly important for environmental regulations. Additionally, absorptive capacity is inter-functional, which can be advantageous to the firm when absorbing and putting to use market knowledge [67]. Institutional factors influence market structures; therefore, a firm’s ability to adapt to institutional constraints through technological and managerial innovation depends on its ability to integrate supply-side organizational and technological knowledge and demand-side customer preferences [61]. Additionally, absorbing spatial information about the business environment is essential for firms to survive [18]. Firms use geographical search methods to learn about external factors such as legal requirements and competitive strategies. Command-and-control regulation is an excellent facilitator for identifying innovation opportunities hampered by organizational inertia and acts as a knowledge-sharing catalyst [52].

Regulation compliance depends on a firm’s knowledge capabilities, which aid in identifying and utilizing accessible knowledge flows [66]. Organizational change and adaptability are continually produced by routines driven by command-and-control regulations [61]. They comprise responses to the environment, but they are also associated with disseminating new information, manufacturing skills, and technology [68]. For instance, managers who see potential in environmental regulations may collaborate with other businesses and build innovative solutions [69]. External knowledge includes new knowledge acquired through absorptive capacity [56].

Because firms may overlook new market prospects due to organizational inertia, environmental regulations may give rise to the integration of external knowledge. In a market-based system, a firm that strives for pragmatic legitimacy will work with others that have unique and complementary skills that they can impart to support its survival and growth [66]. According to Abbas and Sağsan [60], firms are incentivized to attain green goals instead of conventional financial ones in such an institutional framework. This situation will likely continue with the uprise of innovation and environmental regulations [70].

Firms collaborate with their partners to attain practical legitimacy and gain access to crucial and relevant information. Environmental regulations make firms more conscious of resource inefficiencies and potential technological opportunities [71]. Firms want to achieve social objectives and increase profits simultaneously. According to Cai et al. [19], firms pressured for legitimacy are likely to implement green ideas. Additionally, multinational firms will want to legitimize their procedures and activities to sustain credibility with clients and society [34]. Firms with higher green absorptive capacity are more likely to leverage environmental opportunities [68]. Environmental regulations motivate businesses to improve their green initiatives in their processes and products. Thus, increased green absorptive capacity allows firms to effectively recognize, assess, and leverage new market possibilities, leading to the accumulation of external information and the implementation of environmental regulations.

This study has the following hypothesis:

**H5.**  *Green innovation and absorptive capacity are positively associated because of the moderating function of environmental regulations*.

## 3. Sample

This study’s sample is China’s manufacturing industry. Chinese producers profit from the abundance of resources, high rates of output, and increased investment in environmental rehabilitation. The following criteria guided the choice of this sample: First, the nation’s economy and society’s livelihood rely heavily on this primary industry. Second, the industry has seen significant societal challenges, including tax scams, human exploitation, and health and safety-related incidents [72]. Additionally, it is believed that these circumstances will continue unless addressed [73].

According to Jennifer Ho and Taylor [74], the manufacturing industry participates more in social and environmental causes than other industries. It significantly contributes to air, water, waste, and climate change [75]. Previous research [76] verify that manufacturing firms with social activities are shown to have beneficial results. They disclose environmental, production, and social information more frequently than others, facing greater pressure to publish accurate corporate social information.

This study bases its analysis on two stock exchanges under the “A” category—Shanghai and Shenzhen. Manufacturers with listings in both markets are identified. The selection criteria for a representative sample of all A-share listed companies in China from 2010 to 2020 follow. First, the selection excludes financial firms because of accounting comparability matters and environmental issues. Second, it excludes firms with negative net assets, as they are data anomalies. Last, it also excludes firms with unavailable data.

The Industry Classification Guidance for Listed Companies, most recently amended by the China Securities Regulatory Commission, is the foundation for this study’s sample distribution. Patent information and key data are retrieved from firms’ annual reports, the China Stock Market, and the Accounting Research Database. Two hundred and fifty-two (252) firms finalize the selection.

### 3.1. Assessing Variables

#### 3.1.1. Green Innovation

Prior studies reveal that securing accurate variable dimensions in empirical testing produces positive outcomes [77]. In this study, the dependent variable is green innovation. Exclusive right investors are common in green innovation ventures. Business environmental patent applications are examples of green innovation [58]. Firms make patent applications to increase sales, obtain technological advantages, and safeguard their brand and reputation. The best way to assess a firm’s intellectual property activities is to examine its patent filings. Previous studies have utilized environmental patent data to gauge green innovation [58]. In light of these findings, this study supports the direction of policies promoting green innovations based on quantity, such as the volume of environmental patent applications filed by businesses over time.

#### 3.1.2. Absorptive Capacity

Numerous studies estimate absorptive capacity using various measuring techniques. Several investigations have used the number of developers. R&D expenditure can also quantify a firm’s absorptive capacity, as does the number of R&D employees working in R&D departments [41]. Absorptive capacity describes a firm’s capacity to recognize, internalize, and apply new information [25]. This study employs absorptive capacity as its independent variable, dividing R&D expenditure with revenue [25,53].

#### 3.1.3. Board Capital

This study applies two variables—board human capital and board social capital—to precisely examine the influence of board capital [15]. Board capital acts as an independent and moderating variable. The educational background and professional experience of board members determine board human capital (board competence is the size of the board multiplied by the number of directors with prior expertise in economics, accounting, research, engineering, and law). Second, the number of concurrent directorships in other businesses determines board social capital [15].

#### 3.1.4. Environmental Regulation

Environmental issues have led to increased traction in environmental legislation. This study employs environmental regulation as the other independent and moderating variable. A proxy is used to evaluate environmental restrictions: the annual cost of ecological and environmental projects divided by the firm’s revenue [78]; this is consistent with past research.

#### 3.1.5. Control Variables

This study utilizes several control variables to ensure the best outcomes. Firm size, board size, environmental awareness, and sales growth are controllable factors at the corporate governance level. The firm’s regular asset log calculates its size [79]. The number of board members determines board size [29]. Multiplying the number of employees by the sum of the firm’s redesign and greenery-related expenses calculates environmental awareness [77]. Sales growth is the difference between the current year’s sales and previous year’s sales [29].

### 3.2. Empirical Methodology

In statistics and econometrics, longitudinal data and panel data are terms for multi-dimensional data that comprise measurements over time. Observations made for the same participants throughout time are included in a subset of longitudinal data known as panel data [77]. Earlier studies reveal that endogeneity and unobservable heterogeneity problems are frequently linked to panel data [80]. The main issue with panel data is unobservable heterogeneity. Because a firm’s descriptive and projected variables are both endogenous, there is no correlation between them [62]. Therefore, the fixed effect is the most successful at eliminating unobservable heterogeneity [62]. By this assessment, this study’s analysis applies the fixed effect and the random effect. One can use either model depending on the results of the Hausman test [80].

Second, Arellano and Bond [81] created the dynamic panel model, also known as the generalized method model (GMM). This study uses the GMM to address the endogeneity matter. The components’ association evolves with time, and the GMM can effectively overcome this obstacle [80]. Additionally, the GMM can create precise equation evaluations. The delays of expected variables can be leveraged using the GMM to overcome endogeneity in panel data [65]. The GMM manages endogeneity by using “internal adjusting data”. It is also explicit in changing coefficients, making it the most efficient technique for removing endogeneity from panel data [62].

A feasible generalized least squares (FGLS) model further supports the robustness of the empirical findings in this study. When the data demonstrate a substantial residual correlation, the FGLS can evaluate the unidentified outline in a linear regression model [67]. It offers the most effective strategy for handling heteroskedasticity. It is conceivable that the equation’s erroneous terms will be connected in a certain way [62].

### 3.3. Econometric Equations


(1) $$Y_{i,t}=\alpha_{1}+\beta_{1}AC_{1i,t}+\gamma_{1}Z_{i,t}+\mu_{i,t}$$

(2) $$Y_{i,t}=\alpha_{2}+\beta_{2}BHK_{2i,t}+\gamma_{2}Z_{i,t}+\mu_{i,t}$$

(3) $$Y_{i,t}=\alpha_{3}+\beta_{3}BSK_{3i,t}+\gamma_{3}Z_{i,t}+\mu_{i,t}$$

(4) $$Y_{i,t}=\alpha_{4}+\beta_{4}ER_{4i,t}+\gamma_{4}Z_{i,t}+\mu_{i,t}$$

(5) $$Y_{i,t}=\alpha_{5}+\beta_{5}AC_{1i,t}+\beta_{6}BHK_{2i,t}+\beta_{7}AC_{1i,t}*BHK_{2i,t}+\gamma_{5}Z_{i,t}+\mu_{i,t}$$

(6) $$Y_{i,t}=\alpha_{6}+\beta_{8}AC_{1i,t}+\beta_{9}BSK_{3i,t}+\beta_{10}AC_{1i,t}*BSK_{3i,t}+\gamma_{6}Z_{i,t}+\mu_{i,t}$$

(7) $$Y_{i,t}=\alpha_{7}+\beta_{11}AC_{1i,t}+\beta_{12}ER_{3i,t}+\beta_{13}AC_{1i,t}*ER_{4i,t}+\gamma_{7}Z_{i,t}+\mu_{i,t}$$

These seven equations have been developed to empirically probe this study of firms *i* and year *t*. In these equations, $Y_{i,t}$ shows green innovation as an dependent variable of firms *i* at year *t*. $AC_{i,t}$: shows absorptive capacity variable. $BHK_{i,t}$: highlights the board human capital. $BSK_{i,t}$: highlights the board social capital. $ER_{i,t}$: reveals the environmental regulation. $\beta AC_{1i,t}*BHK_{i,t}$: shows the interaction of absorptive capacity and board human capital. $\beta AC_{1i,t}*BSK_{i,t}$: shows the interaction of absorptive capacity and board social capital. $\beta AC_{1i,t}*ER_{i,t}$: shows the interaction of absorptive capacity and environmental regulation. $\gamma_{}Z_{i,t}$: highlights the control variables of firm *i* at year *t*. $\mu_{i,t}$: reveals the error term; $\alpha_{n}$: shows the constant term, *n* = 1; $\beta_{m},\;\gamma_{n}$ shows coefficients to be estimated; and *m* = 1, 2, 3, 4, 5, 6, 7, 8, 9, 10, 11, 12, 13.

## 4. Results

The descriptive statistics and Pearson test for absorptive capacity (AC), green innovation (GI), board capital (BHK and BSK), environmental regulation (ER), and control variables are displayed in Table 1. The values for the mean and standard deviation are shown in this table. The Pearson coefficient correlation analysis reveals a relationship between AC and GI, BHK and GI, BSK and GI, and ER and GI, and then the moderating connection. The majority of the factors exhibit a strong and favorable connection. Every one of the control variables is positively and significantly correlated with the others. For this test, there are three levels of significance: 1%, 5%, and 10%.

Results of the fixed effect and GMM approaches are shown in Table 2 for the correlations amid GI and AC, GI and BHK, GI and BSK, and GI and ER. It is evident that AC has a substantial and positive effect on GI, focusing on model 1 first, which has fixed effect values (β_ = 0.798, *p* = 0.01) and GMM values (β_ = 0.751, *p* = 0.01). Second, model 2 fixed effect values and GMM values (β_ = 4.559, *p* = 0.01), (β_ = 4.656, *p* = 0.01) demonstrate that BHK significantly and satisfactorily influences GI. Third, model 3 fixed effect values and GMM values (β_ = 0.355, *p* = 0.01), (β_ = 0.463, *p* = 0.01) demonstrate that BSK significantly and satisfactorily influences GI. Fourth, model 4 shows that ER significantly and favorably affects GI, with fixed effect values (β_ = 0.114, *p* = 0.01) and GMM values (β_ = 0.062, *p* = 0.01). Table 2 also displays the Hausman test results for models 1 through 4, which support the fixed effect approach over the random one (β_ = 158.39, *p* = 0.01, β_ = 186.42, *p* = 0.01, β _ = 52.66, *p* = 0.01, β_ = 59.07, *p* = 0.01).

Moreover, Table 3 displays the interaction values of board human capital and absorptive capacity in model 1 (AC*BHK) (β_ = 0.246, β_ = 0.973) by means of fixed effect and GMM models; both have a 1% level of significance. Model 2 displays the interaction values of board social capital and absorptive capacity (AC*BSK) (β_ = 0.487, β_ = 0.484) by means of fixed effect and GMM models; both have a 1% level of significance. Table 3 also includes the Hausman test results for models 1–3, which favor the fixed effect technique over the random effect method (β_ = 177.23, *p* = 0.01; β_ = 410.55, *p* = 0.01). To conclude, our findings were in line with Hypothesis 3, which postulated that board capital (social and human) had an impact on the association between absorptive capacity and green innovation.

In addition, model 3 displays the interaction values of environmental regulation and absorptive capacity (AC*ER) (β_ = 1.561, *p* = 0.01, β_ = 3.611, *p* = 0.01) using fixed effect and GMM models. Table 3 also includes the Hausman test results for model 6, which favors the fixed effect technique over the random effect method (β_ = 345.47, *p* = 0.01). To conclude, our results were consistent with Hypothesis 5, which stated that environmental regulation affected the relationship between absorptive capacity and green innovation.

### Reliability of the Results

A robustness test for future investigation was carried out in this study using feasible generalized least squares (FGLS). Researchers assert that issues such as autocorrelation and heteroskedasticity can also be resolved by FGLS. Table 4 reveals the robustness results of the study. Table 4 displays the outcomes of the FGLS technique for the relationships between GI and AC, GI and BHK, GI and BSK, and ER and GI. It is evident that AC has a substantial and positive effect on GI, starting with model 1 (β_ = 0.774, *p* = 0.01). Similarly, model 2 reveals the connection of BHK with GI (β_ = 0.5.523, *p* = 0.01), and model 3 reveals the connection of BSK with GI (β_ = 0.034, *p* = 0.01). Moreover, models 3 and 4 highlight the interaction effect of board human capital and absorptive capacity (AC*BHK) (β_ = 0.452, *p* = 0.01), board social capital and absorptive capacity (AC*BSK) (β_ = 0.667, *p* = 0.01), and environmental regulation and absorptive capacity (AC*ER) (β_ = 2.994, *p* = 0.01). This probe also supported the previous finding of the study.

## 5. Discussion 

This study explores how absorptive capacity affects green innovation. The results reveal that absorptive capacity is crucial for green innovation, which is in line with past studies on the beneficial effects of absorptive capacity on green innovation performance [28,43]. Absorption capacity enables a firm to be more ecologically innovative if it has a proactive plan that recognizes and assesses future environmental changes. A firm’s absorption capacity increases its ability to develop green innovations such as ecologically friendly goods, services, and practices. According to Chen et al. [11], absorptive capacity positively impacts radical, incremental, and service-based green innovation. Firms can employ internal and external knowledge to assume new ecologically friendly techniques. Chinese firms can integrate internal experience with external knowledge as part of a green innovation strategy to succeed in the Chinese market, where environmental standards are pivotal [9].

The next subject of analysis is board capital’s impact on green innovation. This study provides board capital as a board of directors’ human and social capital, investigating its impact on green innovation from its human and social aspects [82]. The results reveal that board capital significantly affects green innovation; prior research supports this [36,48]. With the relevant knowledge, experience, and external connections, boards will likely display better green innovation performance. Boards can play a significant role in firms of emerging countries, encouraging them to adopt socially responsible behavior to grow their operations in the global market [13]. Boards with more substantial human and social capital provide better guidance and supervision because they have the knowledge and experience to understand complex information expediently [36]. Green techniques ought to intrigue board members. Boards must encourage positive social behaviors to achieve long-term success. Particularly, businesses from developing nations rely on green innovation methods in order to compete on the global market.

The following analysis explores the moderating effect of board capital on the association between absorptive capacity and green innovation. The results highlight that board capital positively moderates the relationship between absorptive capacity and green innovation. Through board capital, a firm can acquire external knowledge that it can subsequently use as an internal resource to improve green innovation [82]. Board capital can function as a controlling factor in managing a firm’s research and environmental practices. Board members with good knowledge of environmental programs can lead green innovation initiatives at the organizational level [83]. Board members represent the firm’s governance [84]; their responsibility is to ensure its long-run survival. Knowledgeable and experienced board members can motivate a firm into R&D [13]. These practices will encourage the firm to adopt environmental practices [85]. Therefore, board capital is supposed to be a strong controlling factor for absorptive capacity and green innovation.

This study also examines the role of environmental regulation in green innovation. It reveals that green innovation performance improves with environmental regulation; previous studies provide support [17,51]. Firms incorporate environmental initiatives into their business planning in response to institutional pressure [57]. Institutions significantly influence a firm’s decision-making behaviors and processes by imposing environmental control in a social context [45]. Firms find themselves in the headlines because of contamination issues. Authorities implement various measures to increase the cost of pollution, inevitably boosting green innovation. Environmental regulations compel firms to provide distinctive goods for the market, fostering corporate novelty.

This study’s final analysis investigates the influence of environmental regulation in moderating the association between absorptive capacity and green innovation. Environmental regulations are beneficial for green innovation, and they encourage a firm’s absorptive capacity. Firms must acquire spatial information about their business environment [18]. Porter et al. [63] support environmental regulations for R&D and firm innovation. Environmental regulations pressure firms to adopt environmentally friendly practices [33], making them more competitive. Environmental restrictions become crucial for firms to succeed in a challenging market; hence, restrictions encourage green innovation [86]. Firms in emerging markets can be incentivized to compete globally with environmental initiatives to meet the international market’s quality, environmental, and social requirements.

Environmental regulations help firms to improve their absorptive capacity [56]. Organizational inertia can cause firms to overlook new market opportunities; environmental legislation can trigger them to seek external expertise. Global warming and sustainable development goals coerce Chinese firms in particular to embrace environmentally responsible corporate practices. China’s economy is one of the fastest expansions globally; it faces significant environmental pressure because of it. Therefore, the Chinese government has established obligatory laws, rules, and regulations to enable the industrial sector to participate in the country’s environmental cleanup initiatives [87]. Environmental regulation is imperative for absorptive capacity and green innovation.

## 6. Conclusions

Environmental issues have gained much attention in emerging nations because of the industrial sector’s increased contribution to conservational challenges. Presumably, the growth of the industrial sector is key to a nation’s economic health. This study addresses the question of a firm’s ability to absorb information, affecting the competencies required for improved green innovation performance. This study also discusses how absorptive capacity can explicitly enhance the success of green innovation. Moreover, the impact of board capital and environmental regulation on green innovation is explored in this study, identifying how they moderate the association between absorptive capacity and green innovation. This study targets Chinese manufacturing firms and employs the fixed effect, GMM, and FGLS models to ensure the reliability of the findings. 

This study confirms that absorptive capacity valuable for green innovation. In addition, this study divide board capital into social and human capital. The results state that both social and human capital enhances green innovation. Importantly, the outcomes confirm that board social and human capital moderate the positive link on the connection between absorptive capacity and green innovation. Moreover, this study concludes that environmental regulations also positively affect green innovation. Lastly, this study concludes that environmental regulations also serve as positive moderator on the on the connection between absorptive capacity and green innovation.

### 6.1. Implications

This study has several management-related ramifications. Managers are encouraged to consider its conclusions. First, this study demonstrates that firms’ green innovation strategies are influenced by absorptive capacity. Therefore, absorptive capacity is imperative for improving green innovation at the organizational level. Additionally, absorptive capacity enables firms to respond proactively to institutional demands for green innovation. This study highlights the significance of a board’s human and social capital for green innovation, thus establishing a consummation of the firm’s corporate governance and environmental performance. This study emphasizes how board capital and green innovation affect firms, thus enabling them to meet legal and stakeholder demands for the environment.

Second, the present study recommends considering its implications from the China perspective. The board capital of its listed firms has proven that they improve green innovation and, thus, realize the government’s environmental initiatives. This study suggests that effective board monitoring can strengthen the association between absorptive capacity and green innovation; top management, shareholders, and policymakers must play this role. Therefore, the importance of board capital and other board members may never be overlooked for greater social practices.

Third, this study highlights the importance of environmental regulations and firms’ absorptive capacity for green innovation. Top management can monitor legislative changes and their peers’ current technology strategies. Monitoring regulatory change and predicting technological paths in industries can lessen the uncertainty associated with green innovation and reinforce peer credibility and corporate branding. This study’s findings emphasize the importance of environmental control for long-term sustainability and growth. Governments and policymakers can use this study’s recommendations to stimulate market competitiveness, with participating firms observing progressive environmental practices.

Therefore, this study encourages stakeholders to engage in environmental strategy development with firms; firms in emerging economies are likely to benefit if they seek to compete globally. Environmental and social practices at the corporate level improve brand recognition. CSR measures must be transparent and emphatic, increasing a firm’s profit. Regulatory bodies must ascertain that firms have environmental initiatives supported by top management. This study calls for firms to be incentivized to improve sustainability practices and engage in community development and environmental remediation.

### 6.2. Limitations and Direction

This study has empirical flaws that may point the way for new lines of inquiry. Focusing on China, this study examines the complex relationship between absorptive capacity and green innovation. Future research may focus on other developing countries in comparison to developed countries. The impact of corporate governance, business patterns, and other factors on the relationship between absorptive capacity and green innovation can be sources of evaluation for potential studies focusing on other industries.

## Figures and Tables

**Table 1:** Correlation Test and Descriptive Stats.

Variables	M	SD	1	2	3	4	5	6	7	8	9	10	11	12
1. GI	0.31	0.38	1											
2. AC	0.28	0.38	0.93 ***	1										
3. BHK	0.49	0.73	0.93 ***	0.86 ***	1									
4. BSK	0.85	0.23	0.01	−0.03 *	0.02	1								
5. ER	0.46	0.32	0.24 ***	0.36 ***	0.24 ***	−0.44 ***	1							
6. ACBHK	0.22	0.44	0.75 ***	0.66 ***	0.75 ***	−0.10 ***	0.28 ***	1						
7. ACBSK	0.50	0.84	0.77 ***	0.67 ***	0.83 ***	0.09 ***	0.27 ***	0.71 ***	1					
8. ACER	0.18	0.17	0.78 ***	0.71 ***	0.83 ***	−0.08 ***	0.32 ***	0.97 ***	0.75 ***	1				
9. FS	0.22	0.39	0.54 ***	0.47 ***	0.52 ***	0.06 ***	0.19 ***	0.45 ***	0.50 ***	0.45 ***	1			
10. BS	0.43	0.44	−0.10 ***	−0.09 ***	−0.08 ***	0.07 ***	0.34 ***	−0.02 ***	0.03	−0.02	0.23 ***	1		
11. EA	0.78	0.83	0.13 ***	0.19 ***	0.13 ***	−0.01	0.54 ***	0.10 ***	0.21 ***	0.12 ***	0.47 ***	0.43 ***	1	
12. SG	0.51	0.52	0.76 ***	0.62 ***	0.71 ***	0.02	0.19 ***	0.64 ***	0.63 ***	0.62 ***	0.68 ***	0.17 ***	0.10 ***	1

* *p* < 0.1; *** *p* < 0.01.

**Table 2:** The connection of AC, BHK, BSK, and ER with Green Innovation.

	Model 1		Model 2		Model 3		Model 4	
Variables	GI		GI		GI		GI	
	Fixed Effect	GMM	Fixed Effect	GMM	Fixed Effect	GMM	Fixed Effect	GMM
AC	0.798 ***	0.751 ***						
BHK			4.559 ***	4.656 ***				
BSK					0.355 ***	0.463 ***		
ER							0.114 ***	0.062 ***
FS	−0.034 ***	−0.031 ***	0.005	−0.017 **	0.031 **	−0.012	0.041 ***	0.009
BS	−0.133 ***	−0.190 ***	−0.072 ***	−0.100 ***	−0.285 ***	−0.461 ***	−0.290 ***	−0.465 ***
EA	0.173 ***	0.147 ***	−0.086 **	0.063	0.069	0.322 ***	−0.011	0.288 ***
SG	0.172 ***	0.191 ***	0.120 ***	0.136 ***	0.505 ***	0.563 ***	0.502 ***	0.567 ***
Constant	0.055 ***	0.075 ***	0.056 ***	0.057 ***	0.134 ***	0.104 ***	0.120 ***	0.116 ***
R^2^	0.9177		0.8859		0.6270		0.6596	
F	18.14 ***		20.40 ***		10.17 ***		19.04 ***	
N	3020	2514	3020	2514	3020	2514	3020	2514
Hausman Test	158.39 ***		186.42 ***		52.66 ***		59.07 ***	
Wald Chi^2^		48,786.40 ***		35,871.14 ***		4862.39 ***		4806.49 ***

** *p* < 0.05; *** *p* < 0.01.

**Table 3:** The Interaction Results.

	Model 1		Model 2		Model 3	
Variables	GI		GI		GI	
	Fixed Effect	GMM	Fixed Effect	GMM	Fixed Effect	GMM
AC	0.516 ***	0.489 ***	0.744 ***	0.699 ***	0.758 ***	0.663 ***
BHK	1.982 ***	1.659 ***				
ACBHK	0.246 ***	0.973 ***				
BSK			0.016	−0.001		
ACBSK			0.487 ***	0.484 ***		
ER					0.001	−0.002
ACER					1.561 ***	3.611 ***
FS	−0.022 ***	−0.025 ***	0.030 ***	−0.029 ***	−0.033 ***	−0.030 ***
BS	−0.088 ***	−0.115 ***	−0.115 ***	−0.167 ***	−0.126***	−0.166 ***
EA	0.066 ***	0.085 ***	0.092 **	0.074 **	0.162 ***	0.127 ***
SG	0.114 ***	0.124 ***	0.155 ***	0.175 ***	0.165 ***	0.172 ***
Constant	0.044 ***	0.061 ***	0.051 ***	0.071 ***	0.053 ***	0.077 ***
R^2^	0.9653		0.9428		0.9471	
F	18.73 ***		13.29 ***		14.48 ***	
N	3020	2514	3020	2514	3020	2514
Hausman Test	177.23 ***		410.55 ***		345.47 ***	
Wald Chi^2^		88,138.10 ***		55,495.35 ***		57,204.24 ***

** *p* < 0.05; *** *p* < 0.01.

**Table 4:** The Result of the Robustness.

	Model 1		Model 2		Model 3		Model 4
Variables	GI		GI		GI		GI
	FGLS	FGLS	FGLS	FGLS	FGLS	FGLS	FGLS
AC	0.774 ***				0.548 ***	0.729 ***	0.737 ***
BHK		5.523 ***			2.526 ***		
BSK			0.034 ***			0.015 ***	
ER				0.098 ***			−0.069 ***
ACBHK					0.452 ***		
ACBSK						0.667 ***	
ACER							2.994 ***
FS	−0.007 *	−0.018 ***	0.033 **	0.075 ***	−0.011 ***	−0.024 ***	−0.021 ***
BS	−0.058 ***	−0.042 ***	−0.254 ***	−0.241 ***	−0.024 ***	−0.062 ***	−0.048 ***
EA	0.015 **	0.169 ***	0.742 ***	0.385 ***	0.023 ***	0.051 ***	0.173 ***
SG	0.217 ***	0.072 ***	0.585 ***	0.551 ***	0.079 ***	0.187 ***	0.191 ***
Constant	0.015 ***	0.018 ***	0.037 ***	0.025 ***	0.010 ***	0.010 ***	0.031 ***
N	3020	3020	3020	3020	3020	3020	3020
Wald Chi^2^	8720.64 ***	7279.37 ***	19,972.95 ***	16,962.88 ***	95,686.98 ***	24,171.43 ***	44,954.86 ***

* *p* < 0.1; ** *p* < 0.05; *** *p* < 0.01.

## Data Availability

Not applicable.
